# Editorial: Impact of technology on human behaviors in medical professions education

**DOI:** 10.3389/fmed.2025.1546296

**Published:** 2025-02-07

**Authors:** Muhammad Azeem Ashraf, Samson Maekele Tsegay

**Affiliations:** ^1^Institute of Educational Sciences, Hunan University, Changsha, China; ^2^School of Education, Anglia Ruskin University, Cambridge, United Kingdom

**Keywords:** technology, medical education, medical technology (Med-Tech) innovations, educational technology research, technology in a classroom, technology in medical education

The Research Topic focuses on the impact of technology on human behaviors in medical sciences teaching and learning. It also explores the effect of social, psychological, and other factors on the use of technology in medical education. In so doing, the Research Topic focuses on the integration of technology and the role of human behaviors in improving the teaching and learning process of medical education. Technology has revolutionized education by allowing teachers and students to establish new ways of teaching and learning (1, 2). It has transformed the delivery of knowledge and the behaviors and attitudes of educators and learners. Integrating advanced tools, such as virtual reality (VR), artificial intelligence (AI), and online learning platforms, has improved how medical students and professionals acquire skills, interact with patients, and engage with peers. Interactive technologies like simulation-based learning and VR allow students to engage in realistic scenarios without risk to real patients. Digital tools such as shared virtual workspaces and cloud-based platforms enable students to collaborate on projects regardless of their geographical location. Digital platforms such as Massive Open Online Courses (MOOCs) and online medical databases provide professionals with instant access to the latest research and clinical guidelines. This accessibility promotes continuous learning habits among medical practitioners, encouraging them to stay updated and adapt to evolving practices.

Technology has also impacted collaboration in medical education. This fosters teamwork and communication skills that are essential in healthcare settings. Moreover, technology encourages interdisciplinary learning, as students from different fields can easily participate in joint training sessions, enhancing their ability to work in diverse teams. In addition to its support in improving teaching and learning in medical education, technology has raised ethical questions that shape behaviors. For example, in the Research Topic, Alam et al. argued that, despite AI's immense potential for advancing healthcare and medicine, careful attention must be paid to ethical considerations. This suggests that using AI in diagnosis and treatment planning requires students to learn about data privacy, algorithm bias, and ethical decision-making. Ethical considerations associated with healthy/unhealthy use of technology in medical sciences are also related to human behavior. Hence, medical education must incorporate knowledge, skills, and attitudes to prepare students to navigate complex ethical landscapes.

The Research Topic consists of 11 articles examining technology's impact on medical education from different perspectivesThe articles included in the Research Topic can be categorized into three themes.

1. Digital identity and professionalism in medical profession education

The articles on this theme discuss the impact of technology on human behavior, especially the connection between technology and digital professionalism and psychological changes. Guraya et al., explored the modeling of digital identity and virtual engagement in the medical field, emphasizing mission-driven e-professionalism. They identified three key components: solidification, digitally cultural fitness, and shared agency. Moreover, Kitamura et al. used text mining techniques that analyze qualitative information with quantitative features to investigate how rehabilitation students' goals change during their first year at university. Overall, the articles highlight how technology affects the ethical identity and psychological changes of students.

2. Technology and instructional methods in medical profession education

Considering the multifaceted impact of technology on human behaviors in medical professions education, offering both opportunities and challenges, the Research Topic included articles that contribute to advancing knowledge on how technology impacts medical education through instructional methods. Delafontaine et al. conducted an observational retrospective monocentric study in a French physiotherapy school to examine the consequences of a blended learning program for musculoskeletal anatomy on student skills. Schievano et al. examined the effectiveness of a blended e-learning program, via the PhArmacoVIgilance Africa (PAVIA) training program, and its adaptation during the COVID-19 pandemic. Ashraf et al. examined the role of blended learning in improving medical students' academic performance through self-regulatory learning and technological competence. Chen et al. investigated the application effect of the online and offline mixed teaching mode in nursing practice teaching based on the Source Message Channel Receiver (SMCR) communication model. Sadiq et al. researched integrating technology-enhanced learning in medical education by introducing an E-Portal training program to allow health professions educators to learn essential skills for proficiently employing digital tools in instruction. The articles share the idea that technology affects the experiences of medical students, teachers, and professionals and thus students' learning.

3. Artificial intelligence in medical profession education

The articles of the Research Topic in this theme explore Artificial Intelligence in medical education, which helps to create awareness for the healthy use of technology. Alam et al. advocated the integration of AI tools in medical education, specifically learner-oriented AI tools, and proposed guidelines for medical students to use these tools. Wang et al. studied the relationship between social media-driven networks and job performance among primary healthcare professionals, highlighting knowledge sharing as a mediating factor. Ismail et al. explored non-verbal communication techniques (NVC) during online feedback sessions for communication skill activities in a medical education module and indicated its impact on conveying nuanced information. Du et al. proposed the ensemble model to show the role of AI in disease prediction for medical practitioners.

In conclusion, this Research Topic has explored the impact of technology on human behavior in medical professions education from various perspectives using different theoretical frameworks. The findings suggest that while technology fosters engagement, collaboration, and lifelong learning, it also requires a concerted effort to preserve essential human skills and attitudes, such as empathy and ethical reasoning. By embracing a balanced approach, medical educators can ensure that technology enhances, rather than diminishes, the humanistic aspects of medical practice. On a different note, it is important to question: how might technology impact medical professions education differently from other fields or disciplines?
